# 25-year follow-up of treated and not-treated adolescents after the Spitak earthquake: course and predictors of PTSD and depression

**DOI:** 10.1017/S0033291719003891

**Published:** 2021-04

**Authors:** Armen K. Goenjian, Alan M. Steinberg, David Walling, Sheryl Bishop, Ida Karayan, Robert Pynoos

**Affiliations:** 1 Department of Psychiatry and Biobehavioral Sciences, UCLA/Duke University National Center for Child Traumatic Stress, University of California, Los Angeles, Los Angeles, CA, USA; 2 Collaborative Neuroscience Network, Garden Grove, CA, USA; 3 Psychiatric Outreach Program, Armenian Relief Society, Long Beach, CA, USA; 4 School of Nursing, University of Texas Medical Branch at Galveston, Galveston, TX, USA

**Keywords:** Depression, disaster, earthquake, longitudinal study, PTSD, risk and protective factors

## Abstract

**Background:**

There is a paucity of long-term prospective disaster studies of the psychological sequelae among survivors.

**Methods:**

At 1½ and 25 years after the Spitak earthquake, 142 early adolescents from two cities were assessed: Gumri (moderate–severe exposure) and Spitak (very severe exposure). The Gumri group included treated and not-treated subjects, while the Spitak group included not-treated subjects. Instruments included: DSM-III-R PTSD-Reaction Index (PTSD-RI); DSM-5 PTSD-Checklist (PCL); Depression Self-Rating Scale (DSRS); and Center for Epidemiological Studies-Depression Scale (CES-D).

**Results:**

(1) Between 1½ and 25 years, PTSD rates and mean scores decreased significantly in the three groups (over 50%). However, at 25 years 9.1–22.4% met DSM-5 PTSD criteria. (2) At 1½ years, the Spitak group had higher PTSD-RI (*p* < 0.001) and DSRS scores (*p* < 0.001) compared to the Gumri-not-treated group. At 25 years, the Spitak group that had experienced fewer post-earthquake adversities (*p* < 0.03), had a greater decrease in PTSD-RI scores (*p* < 0.02), and lower CES-D scores (*p* < 0.01). (3) Before treatment, PTSD-RI and DSRS scores did not differ between the Gumri-treated and not-treated groups. At 25-years, the Gumri-treated group showed a greater decrease in PTSD-RI scores (*p* < 0.03), and lower mean PTSD-RI (*p* < 0.02), PCL (*p* < 0.02), and CES-D (*p* < 0.01) scores. (4) Predictors of PTSD symptom severity at 25-years included: home destruction, treatment, social support, post-earthquake adversities, and chronic medical illnesses.

**Conclusion:**

Post-disaster PTSD and depressive symptoms can persist for decades. Trauma-focused treatment, alleviation of post-disaster adversities, improving the social ecology, and monitoring for chronic medical illnesses are essential components of recovery programs.

## Introduction

The field of Disaster Psychiatry has become well-established and is anchored in numerous studies of the psychological sequelae among survivors of various types of natural and man-made disasters. The majority of disaster studies conducted among children and adolescent do not extend past 12 months (Terasaka, Tachibana, Okuyama, & Igarashi, 2015; Wang, Chan, & Ho, 2013). Consequently, there is a lack of information on the natural course and predictors of the psychological sequelae past a decade due to difficulties inherent in conducting longer-term post-disaster research. Reviews of posttraumatic stress disorder (PTSD) studies among children and adolescents exposed to disasters have shown that only about 10% of studies have extended beyond 3 years (Terasaka et al., 2015; Wang et al., 2013). Some of these studies have been cross-sectional (Agustini, Asniar, & Matsuo, 2011; Goenjian et al., 2009; Jia et al., 2013; Karakaya, Ağaoğlu, Coskun, Sişmanlar, & Yildiz Oc, 2004; Morgan, Scourfield, Williams, Jasper, & Lewis, 2003; Thordardottir et al., 2016), while others have been longitudinal (Goenjian et al., 2005; Green et al., 1994; Kronenberg et al., 2010; McFarlane & Van Hooff, 2009; Najarian, Sunday, Labruna, & Barry, 2011; Piyasil et al., 2011; Sahin, Batigün, & Yilmaz, 2007; Udwin, Boyle, Yule, Bolton, & O'Ryan, 2000; Ularntinon et al., 2008; Yule et al., 2000). Of these studies, four were conducted past a decade post-disaster (Green et al., 1994; McFarlane & Van Hooff, 2009; Morgan et al., 2003; Najarian et al., 2011). Three showed that PTSD was persistent in adulthood among a substantial proportion (7% to 29%) of child and adolescent survivors (Green et al., 1994; Morgan et al., 2003; Najarian et al., 2011), while the fourth found a small direct effect of the disaster on adult psychiatric morbidity at follow-up (McFarlane & Van Hooff, 2009).

Risk factors for PTSD identified in these medium to longer-term studies have included: death toll and proximity to the event (Furr, Corner, Edmunds, & Kendall, 2010); severity of initial exposure (Piyasil et al., 2007); previous history of trauma and physical injury (Ularntinon et al., 2008); occurrence of subsequent traumatic events (Perkonigg et al., 2005); pre-disaster psychosocial and physical difficulties in childhood (Udwin et al., 2000); and lack of social support in the aftermath (Thordardottir et al., 2016).

Regarding treatment outcome studies of PTSD among children and adolescents after disasters, most follow-ups have been conducted within 6–12 months (Chemtob, Nakashima, & Hamada, 2002; March, Amaya-Jackson, Murray, & Schulte, 1998; Pityaratstian et al., 2015). To our knowledge, only a few studies that have investigated post-disaster treatment effect have employed longer (up to 4 years) follow-up periods (Basoglu, Salcioglu, & Livanou, 2007; Giannopoulou, Dikaiakou, & Yule, 2006; Goenjian et al., 1997, 2005). These studies have shown a beneficial effect of a variety of trauma-focused interventions in reducing the severity of PTSD symptoms.

### The Spitak earthquake

In 1988, a catastrophic earthquake struck Armenia with a death toll estimated to be over 25 000. In Spitak, located 6.6 miles from the epicenter (population 30 000), the intensity of the earthquake was rated X (‘Devastating’) on the Medvedev–Sponheuer–Karnik-64 (MSK-64) scale, with a death toll of 5000. In Gumri, located 16.8 miles from the epicenter (population 250 000), the intensity of the earthquake was rated IX (‘Destructive’), with a death toll of 17 000 (Balassanian et al., 1995).

Due to the pervasive mental health problems related to the disaster and lack of adequate mental health workers in the earthquake zone, the Psychiatric Outreach Program was initiated by volunteer mental health workers from the United States in collaboration with the Ministries of Education and Health of Armenia, and the Armenian Relief Society of the Western United States. The purpose was to provide mental health services to the survivors. Initially, the work was focused on schools, and later in clinics. During the two decades following the earthquake, periodic assessments were made to monitor the mental health recovery of students who were being followed. The current study is a 25-year post-earthquake follow-up of a cohort evaluated at 1½ years post-earthquake (Goenjian et al., 1995). The purpose was to (1) assess the course of earthquake-related PTSD and depressive symptoms among differentially exposed subjects in Spitak and Gumri; (2) assess the effect of trauma-grief focused treatment; and (3) investigate potential risk and protective factors for PTSD and depression.

## Methods

### Participants

In 1990, 1½ years after the Spitak earthquake, 164 early adolescents aged 12–14 years from Gumri (*N* = 94) and Spitak (*N* = 70) were evaluated for PTSD, depression, and separation anxiety disorder. Information on these subjects has been reported previously (Goenjian et al., 1995). Within a few weeks after the assessment, school-based trauma/grief-focused brief psychotherapy was provided in some schools in Gumri and not others due to the shortage of qualified mental health workers. The selection of schools was based on proximity to the location of the clinical staff, availability of transportation to the schools, and adequacy of working space at the schools. These schools were representative of the schools in the region. The destruction and death toll was widespread throughout the city so that the children across Gumri schools experienced similar levels of earthquake exposure. All students in the classrooms selected for the intervention participated.

Data for the current study were collected in 2014, 25-years post-earthquake. Seventy-six of the original 94 participants from Gumri were located, with only one refused to participate. Three of the original group had died, and the rest were lost to follow-up. Of these 75 participants, 33 were among the group of 36 students who were provided treatment at 1½ years shortly after the baseline assessments. The rest (*N* = 42) had not received mental health treatment up to the time of the follow-up. In Spitak, 67 of the original 70 subjects were located and completed the study. None of these subjects had received mental health treatment since the earthquake. Two of the original group had died, and the third was lost to follow-up. The total number of participants in 2014 was 142 (87% of the original cohort). The high rate of capturing subjects at follow-up was because many of the original participants had remained in the city they lived in at the time of the earthquake, were cooperative in helping to locate their peers from the original cohort, and were positive about participating.

After obtaining approval from the IRB Company (a fully accredited IRB by the Association for the Accreditation of Human Research Protection Programs), the study subjects were given a full description of the study, including potential risks and benefits. Written informed consent was obtained from all participants.

### Treatment at 1½ years post-earthquake

The treatment provided at 1½ years post-earthquake, after the baseline data were collected, has been detailed previously (Goenjian et al., 2005). It consisted of four 90-min group therapy sessions with all the students in the same classroom and an average of two 1-hour individual therapy sessions over 3 weeks. It targeted five major areas: traumatic experiences; trauma reminders; losses and grief; secondary adversities; and developmental issues. The treatment included psychoeducation and interpersonal and cognitive-behavior therapy techniques. No medications were prescribed.

### Instruments

Demographic information collected at 25 years (Tables 1 and 2) included data on *City During the Earthquake*; *Age*; *Sex*; *Current Employment*; *Education*; and *Marital Status*. *Current Employment* was dichotomized: employed (reference group), and unemployed. *Education* was dichotomized: ⩽high school (reference group), and >high school education. *Marital status* was dichotomized: married (reference group), and separated, divorced, single, widowed.

**Table 1.** Demographic, earthquake exposure and clinical characteristics of not-treated differentially exposed subjects from Spitak and Gumri at 1½ and 25 years after the 1988 Spitak earthquake

Characteristics	Spitak-not-treated (*N* = 67)		Gumri-not-treated (*N* = 42)		
	*M* or *n*	s.d. or %	*M* or *n*	s.d. or %	*t*(df) or χ^2^; *p*
*Demographic variables*					
Age (*M*, s.d.)	37.76	0.88	38.24	1.97	1.49 (51.47); 0.14
Sex (*n*, %)					
Male	27	40.3	14	33.3	0.53; 0.46
Female	40	59.7	28	66.7	
Current employment (*n*, %)					
Yes	40	59.7	23	54.8	0.26; 0.61
No	27	40.3	19	45.2	
Education (*n*, %)					
⩽High school	27	40.3	11	26.2	2.26; 0.13
>High school	40	59.7	31	73.8	
Marital status (*n*, %)					
Married	57	85.1	32	76.2	1.36; 0.24
Separated, divorced, single, widowed	10	14.9	10	23.8	
*Pre-EQ trauma and EQ related variables*					
Pre-EQ trauma (*n*, %)					
Yes	18	26.9	8	28.6	0.87; 0.35
No	49	73.1	34	71.4	
EQ intensity on MSK-64 Scale (Roman numerals)	X		IX		
	‘Devastating’		‘Destructive’		
Home destruction (EQ related) (*n*, %)	56	83.6	21	50	14.04; <**0.001**
None to moderate home damage	11	16.4	21	50	
Nuclear family death (EQ related) (*n*, %)					
Yes	25	37.3	12	28.6	0.88; 0.34
No	42	62.7	30	71.4	
Serious injury (EQ related) (*n*, %)					
Yes	15	23.1	1	2.4	8.59; <**0.01**
No	50	76.9	41	97.6	
Scared during the EQ (*n*, %)					
None/moderate	11	16.4	10	23.8	0.91; 0.34
A lot/very much	56	83.6	32	76.2	
*Intervening variables*					
Post-EQ traumas (*n*, %)					
Yes	26	38.8	23	54.8	2.66; 0.10
No	41	61.2	19	45.2	
Adversities (post-EQ) (*n*, %)					
No difference/better	18	26.9	4	9.5	4.82; <**0.03**
Worse	49	73.1	38	90.5	
Change SES (post-EQ) (*n*, %)					
No difference/better	32	47.8	23	54.8	0.50; 0.48
Worse	35	52.2	19	45.2	
Chronic illnesses since the EQ (*M*, s.d.)	1.85	1.54	1.76	1.57	−0.29 (107); 0.77
Social support score, current (*M*, s.d.)	28.0.	7.7	25.8	9.1	−1.35 (107); 0.18
*1½ and 25 year clinical measures*					
1½ year PTSD-RI-based Dx (*n*, %)	37	55.2^a^	15	35.7^b^	3.93; **<0.05**
25 year PTSD-RI-based Dx (*n*, %)	19	28.3^a^	6	14.3^b^	2.89; <0.09
1½ year SAD symptom count (*M*, s.d.)	6.1	2.1	5.8	2.4	−0.78 (101); 0.44
1½ year DSRS score (*M*, s.d.)	19.0	4.0	15.2	5.0	−4.19(102); <**0.001**
25 year CES-D score (*M*, s.d.)	13.6	10.7	20.7	12.9	2.12, (107); <**0.01**
25 year PCL scores (*M*, s.d.)	31.5	14.1	33.2	15.2	0.59, (107), 0.55
25 year PCL-PTSD Dx (*n*, %)	15	22.4	7	16.6	0.52; 0.47

EQ, earthquake; Dx, diagnosis; MSK-64, Medvedev–Sponheuer–Karnik-64 scale; PTSD-RI, Posttraumatic Stress Disorder-Reaction Index; PCL, PTSD Checklist; DSRS, Depression Self Rating Scale; CES-D, Center for Epidemiologic Studies Depression Scale; SAD, separation anxiety disorder.

a Spitak-not-treated group by time (1½ and 25 years) comparison of PTSD rates: 55.2%^a^ to 28.3%^a^ (χ^2^ = 9.93, *p* < 0.002).

b Gumri-not-treated group by time (1½ and 25 years) comparison of PTSD rates: 35.7%^b^ to 14.3%^b^ (χ^2^ *=* 5.14, *p* < 0.02).

**Table 2.** Demographic, earthquake exposure, and clinical characteristics of Gumri-treated *v.* Gumri-not-treated groups at 1½ and 25 years after the 1988 Spitak earthquake

Characteristic	Gumri-treated (*N* = 33)		Gumri-not-treated (*N* = 42)		
	*M* or *n*	s.d. or %	*M* or *n*	s.d. or %	*t*(df) or χ^2^; *p*
*Demographic variables*					
Age (*M*, s.d.)	36.18	0.950	38.24	1.9	5.93 (61.88); **<0.01**
*Sex* (*n*, %)					
Male	11	33.3	14	33.3	0; 1
Female	22	66.7	28	66.7	
Current employment (*n*, %)					
Yes	16	48.5	23	54.8	0.29; 0.59
No	17	51.5	19	45.2	
Education (*n*, %)					
⩽High school	11	33.3	11	26.2	0.45; 0.50
>High school	22	66.7	31	73.8	
Marital Status (*n*, %)					
Married	29	87.9	32	76.2	1.63; 0.197
Separated, divorced, single, widowed	4	12.1	10	23.8	
*Pre-EQ trauma and EQ-related variables*					
Pre-EQ trauma (*n*, %)					
Yes	5	15.2	8	19.0	0.19; 0.65
No	28	84.8	34	80.9	
EQ Intensity based on MSK-64 Scale (Roman numerals)	IX		IX		
	‘Destructive’		‘Destructive’		
Home – destruction (EQ related) (*n*, %)	13	39.4	21	50	0.83; 0.36
None to moderate home damage	20	60.6	21	50	
Death, EQ related (*n*, %)					
Yes	9	27.3	12	28.6	0.01; 0.90
No	24	72.7	30	71.4	
Serious injury, EQ related (*n*, %)					
Yes	3	9.1	1	2.4	0.31*^α^* (ns)
No	30	90.9	41	97.6	
Scared during the EQ (*n*, %)					
None/moderate	8	24.2	10	23.8	0; 0.97
A lot/very much	25	75.8	32	76.2	
*Intervening variables*					
Post-EQ trauma (*n*, %)					
Yes	14	42.4	23	54.8	1.12; 0.29
No	19	57.6	19	45.2	
Adversities post-EQ (*n*, %)					
No difference/better	4	12.1	4	9.5	0.72*^α^* (ns)
Worse	29	87.9	38	90.5	
Change SES post-EQ (*n*, %)					
No difference/better	23	69.7	25	59.5	0.83; 0.36
Worse	10	30.3	17	40.5	
Chronic illness since EQ (*M*, s.d.)	1.61	1.39	1.76	1.57	−0.45 (73); 0.66
Social support score, current (*M*, s.d.)	27.36	7.34	25.79	9.12	0.81 (73); 0.42
*1½ and 25 year clinical measures*					
1½ year PTSD-RI-based Dx (*n*, %)	11	33.3^a^	15	35.7^b^	0.05, 0.83
25 year PTSD-RI-based Dx (*n*, %)	4	12.1^a^	6	14.3^b^	0.07; 0.78
1½ year SAD symptom count (*M,* s.d.)	6.4	2.4	5.8	2.4	0.96 (73); 0.34
1½ year DSRS score (*M*, s.d.)	16.8	5.1	15.2	5.0	1.28 (72); 0.20
25 year CES-D score (*M*, s.d.)	12.6	10.0	20.7	12.9	−3.07(72.9); <**0.01**
25 year PCL score (*M*, s.d.)	25.2	13.1	33.2	15.2	−2.44 (73); **<0.02**
25 year PCL-based PTSD Dx (*n*, %)	3	9.1%	7	16.6	0.92; 0.34

EQ, earthquake; Dx, diagnosis; MSK-64, Medvedev–Sponheuer–Karnik-64 scale; PTSD-RI, Posttraumatic Stress Disorder-Reaction Index; PCL, PTSD Checklist; DSRS, Depression Self Rating Scale; CES-D, Center for Epidemiologic Studies Depression Scale; SAD, Separation anxiety disorder.

*α* = Fisher's exact test.

a Gumri-treated group by time (1½ and 25 years) comparison of PTSD rates: 33.3%^a^ to 12.1%^a^ (χ^2^ *=* 4.22, *p* < 0.04).

b Gumri-not-treated group by time (1½ and 25 years) comparison of PTSD rates: 35.7%^b^ to 14.3%^b^ (χ^2^ *=* 5.14, *p* < 0.02).

### Earthquake-related variables

City-wide official measures of exposure included the intensity of the earthquake and the death toll attributed to the earthquake. At 25-year follow-up the *UCLA Exposure Questionnaire* was used (Roussos et al., 2005) to measure objective and subjective features of experiences during the earthquake. *Home Destruction* due to the earthquake was dichotomized: none to moderate damage (reference group), and severe damage beyond repair. *Nuclear Family Deaths* related to the earthquake was dichotomized as none (reference group), and ⩾1. *Earthquake-related Serious Injuries* (e.g. crush injuries, fracture, and injuries requiring emergency medical care) were dichotomized: none, mild (reference group), and ⩾1 serious injury. *Subjective Experiences* (i.e. scared during the earthquake), was rated on a 5-point scale: 0 = none, 1 = a little, 2 = moderate, 3 = a lot, and 4 = very much. These scores were dichotomized: none, a little, moderate (reference group), and a lot, very much. *Exposure to Pre- and Post-Earthquake Traumatic Experiences* (events that involved an actual threat of death or serious injury of self or others) were dichotomized absent = none, mild (reference group), and present = moderate to extremely stressful.

*Post-earthquake Adversities* were derived by comparing post-earthquake to pre-earthquake conditions (heating; electricity; food; living space; and transportation). Responses were dichotomized into no difference, better (reference group), and worse. Change of *Socioeconomic Status* (SES) for the 10 years post-earthquake compared to pre-earthquake was dichotomized: no difference, better (reference group), and worse. *Post-earthquake Chronic Illnesses* were self-reported based on the information provided to the subjects at the polyclinics where they were enrolled. The illnesses included hypertension, myocardial infarction, stroke, asthma, and other respiratory illnesses, autoimmune illnesses, chronic genitourinary and digestive tract disease, diabetes, arthritis, musculoskeletal disorders, migraine, and cancer. The total number of illnesses (range: 0–13) for each participant was used for the analysis. Perceived *Current Social Support* by family and friends was assessed using the Duke-UNC Functional Social Support Questionnaire (Broadhead, Gehlbach, de Gruy, & Kaplan, 1988). Responses were scored on a 5-point scale: 1 = ‘much less than I would like’ to 5 = ‘as much as I would like.’ The total score was used for analysis (higher scores reflecting greater perceived social support).

### PTSD and depression measures

Continuous and binary measures for PTSD were used for different reasons. The continuous scale scores provided a more sensitive representation of the progression of symptoms compared to binary measures, i.e. rates meeting diagnostic criteria, while rates were deemed useful for cross-comparisons with other studies that used rates as a primary outcome.

To compare the participants' PTSD severity at two widely separated time points, the same instrument was used at 1½ years (the DSM-III-R based PTSD Reaction Index) and at 25 years. Psychometric properties of the instrument have been reported elsewhere (Steinberg, Brymer, Decker, & Pynoos, 2004). For the PTSD questionnaires, the index trauma was the Spitak earthquake. Frequency of symptom occurrence during the previous month was rated on a 5-point Likert scale: 0 = not at all to 4 = almost every day. Symptom scores of 2 (symptom occurring one to two times a week) to 4 (almost every day), were used to define symptom presence. In a prior report (Pynoos et al., 1993), a cut-off of ⩾40 on the PTSD-RI was used to detect a diagnosis of PTSD. For the current study, more stringent criteria were used. Diagnosis of PTSD for both periods was based on a symptom cluster method (a minimum of 1B, 3C, and 2D category symptoms), as has been done in numerous prior studies on PTSD (McDonald & Calhoun, 2010). As a result, the baseline rates reported in this study are lower than rates reported previously (Goenjian et al., 1995; Pynoos et al., 1993).

At the 25-year follow-up, the Armenian version of the PTSD Check List (PCL) based on DSM-5 criteria (Demirchyan, Goenjian, & Khachadourian, 2015) was also used to estimate the current rate of PTSD. This instrument is a modified version of the validated DSM-IV PCL questionnaire (Movsisyan et al., 2016). As with the PTSD-RI, participants were asked to answer the symptom questions related to the earthquake. Items were rated on a 5-point scale: 0 = not at all; 1 = a little bit; 2 = moderately; 3 = quite a bit; 4 = extremely. To estimate a diagnosis of PTSD on the DSM-5 based PCL questionnaire, the symptom cluster method was used. Ratings of ‘moderately’ to ‘extremely’ were used to define symptom presence. Diagnosis of PTSD was derived by the presence of at least 1B, 1C, 2D, 2E, and category G criteria (functional impairment). Functional impairment was assessed using the *Sheehan Disability Scale* (Leon, Olfson, Portera, Farber, & Sheehan, 1997). Items were rated on a 5-point scale: 0 = not at all; 1 = mildly; 2 = moderately; 3 = markedly; 4 = extremely. A rating of moderate or higher was indicative of impairment.

Depression at 1½ years was evaluated by using the 21-item Depression Self-Rating Scale (DSRS) designed for use in children and adolescents (Asarnow & Carlson, 1985). The ratings of symptom frequency over the previous 2 weeks were made on a 3-point scale: 0 = never; 1 = sometimes; 3 = most of the time. Mean total scores were used for analysis. At follow-up, depression was assessed using the modified validated Armenian version of the Center for Epidemiological Studies Depression Scale (CES-D) (Demirchyan, Petrosyan, & Thompson, 2011). Items were rated on a 4-point scale: 0 = none or rarely, 1 = some of the time; 2 = moderate amount of time; 3 = most or all of the time. Mean total scores were used for analyses.

Translation of the PTSD-RI and DSRS into Armenian followed previously published guidelines, including the use of independent back-translation and pretesting with bilingual subjects (Brislin, 1976). The PCL-C and CES-D were translated into Armenian after several rounds of forward and backward translations until a full concordance between the translation and the original instruments were reached, followed by rigorous testing among bilinguals (Demirchyan et al., 2011, 2015). It remains an important question as to whether, over the many years between administrations of the same instruments, subjects' understanding of some of the terms used may have differed. All of the clinical instruments were administered in person by trained mental health professionals.

## Data analysis

Data were analyzed using Statistical Package for the Social Sciences (SPSS) software (Version 22). Significance was calculated at *p* ⩽ 0.05. All data were examined for normality and homogeneity. χ^2^ analyses were used to explore distributions across nominal variables and to identify appropriate groupings for collapsing categories into dichotomous variables due to small subgroup sample sizes. Tests of differences, (e.g. *t* tests, ANOVA/ANCOVA) were conducted with two-tailed significance (*p* ⩽ 0.05). Post-hoc analyses were conducted using Tukey's HSD, and Scheffe's as well as Tamhane's T2 where issues of heterogeneity were present.

Different variables were dichotomized for different reasons. Ordinal variables, including *Home Destruction*, *Subjective Experience* (*scared during the earthquake*), *Socioeconomic Status*, *Lifetime Traumatic Events*, and *Post-earthquake Adversities*, were dichotomized for use as dummy variables in the multiple regression analyses instead of creating dummy variables for each ordinal category which would have proliferated the variables in the models given and reduced the already limited sample sizes. Numerical variables, including *Nuclear Family Death*(*s*) and *Serious Injuries*, were also dichotomized due to the severely restricted range, as there were only a few subjects with multiple deaths and serious injuries. It was deemed more important to explore differences in qualitative dimensions (e.g. deaths *v.* no deaths) rather than highly skewed and limited continuous distributions.

For this exploratory study, stepwise backward multiple regression analysis was used as it is more inclusive and allows variables that are marginally significant individually but work synergistically as a set, to be explored with the targeted dependent variables. The pre-, peri-, and post-earthquake variables were explored as potential predictors of PTSD-RI, PCL, and CES-D scores at 25 years. Limited sample size precluded assessing all variables of interest in the same model. The variables used included: *Sex*, *Education*, *Home Destruction*, *Nuclear Family Deaths*, *Earthquake-related Serious Injury*, *Post-earthquake Adversities*, *Treatment after Baseline*, *Post-earthquake Traumatic Events*, *PTSD-RI*, *DSRS*, and *SAD* scores at 1½ years, *Post-earthquake Chronic Illnesses*, and *Current Social Support*. Since the risks-based regression coefficients in the final stepwise model err on the high side, the full model results are also reported to provide parameter estimates of all variables considered.

Regarding missing subjects at 25-year follow-up, comparisons of the 1½ year data across the two key outcome variables, PTSD-RI and DSRS scores for those who did not participate in the 25-year follow-up with the data for those who completed the 25-year study yielded no significant differences. This was the case for the Gumri groups combined and the Spitak group, the Gumri-treated and Gumri-not-treated groups, and the Gumri-not-treated and Spitak-not-treated groups. The lack of significant differences at any level supported the decision to drop the missing subjects from the analyses.

The data were checked for completeness. At the 25-year follow-up, there were no missing data. For outcome questionnaires at 1½ years, those missing <10% of the responses, mean values were added in place of the missing data. For those with ⩾10% missing values, the subject questionnaire was excluded from the analysis. For the 1½ year study, a total of six participants had ⩾10% missing data. Four of the six participants had their DSRS and SAD responses excluded. Of the other two, one's DSRS, and another's SAD were excluded.

## Results

### Differentially exposed Gumri-not-treated (moderate–severe exposure) and Spitak-not-treated (very severe exposure) groups

Table 1 shows the demographic characteristics, exposure-related variables, and clinical measures between the differentially exposed Gumri-not-treated and the Spitak-not-treated groups. At 1½ years, between-city comparisons showed significantly higher rates in Spitak (the higher exposure group) for the following: *Home Destruction*, *Earthquake-related Serious Injuries*, and *Post-earthquake Adversities*.

#### PTSD rates at 1½ and 25 years

Table 1 also shows, between 1½ and 25 years, within group comparisons of PTSD rates (based on PTSD-RI). The rates decreased significantly for both the Spitak-not-treated group (55.2% to 28.3%; χ^2^ = 9.94, *p* < 0.002), and the Gumri-not-treated group (35.7% to 14.3%, χ^2^ = 5.14, *p* < 0.02).

Between-group comparisons of PTSD-RI-based PTSD rates at 1½ years showed a significantly higher rate for Spitak (*p* < 0.05). At 25 years, the rate was higher for the Spitak group (28.3%) compared to the Gumri-not-treated group (14.3%), but the difference was not significant (*p* < 0.09). Likewise, at 25 years, there was no significant difference between DSM-5 PCL-based PTSD rates.

#### PTSD severity scores (mean PTSD-RI and delta PTSD-RI scores, and mean PCL scores

**Fig. 1.**
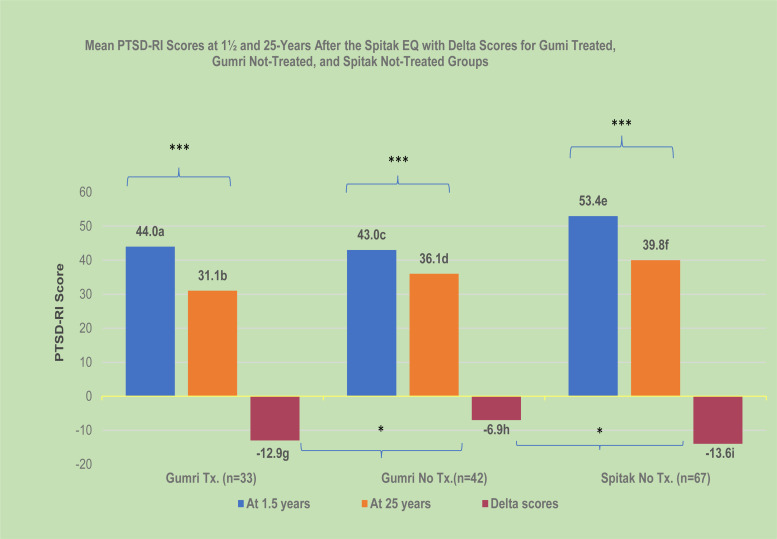
Mean PTSD-RI scores at 1½ and 25 years and the delta scores for the three groups after the 1988 Spitak earthquake. *p < 0.05; ***p < 0.001. PTSD-RI, Posttraumatic Stress Disorder-Reaction Index; Tx, treatment; EQ, earthquake. •A 1½ year comparison between Gumri-treated (a) and Gumri-not-treated (b) groups showed no significant difference. However, at 25 years, Gumri-treated (b) was significantly less than Gumri-not-treated (d) (*t* = −2.03, df = 73, *p* < 0.05).•A 1½ year comparison between differentially exposed group scores showed Spitak-not-treated (e) was higher than Gumri-not-treated (c) (*t* = 6.14, df = 107, *p* < 0.001). At 25 years there was no significant difference between the two groups, even though the mean score for Spitak was higher (*f* > *d*) (*t* = 1.43, df = 107, *p* = 0.15).•Within group by time analysis showed a significant decrease of PTSD-RI scores for the three groups between 1½ and 25 years: Gumri-treated (a > b) (*t* = 6.0, df = 32, *p* < 0.001); (b) Gumri-not-treated (c > d) (*t* = 4.0, df = 41, *p* < 0.001); (c) Spitak-not-treated (e > f) (*t* = 7.05, df = 66, *p* < 0.001).•Between group comparisons of the delta in PTSD-RI scores: Gumri-treated (g) greater than Gumri-not-treated (h) (*t* = 2.23, df = 73, *p* < 0.03); Spitak-not-treated (i) greater than Gumri-not-treated (h) (*t* = 2.42, df = 107, *p* < 0.02).

Figure 1 shows the mean PTSD-RI scores at 1½ and 25 years between the differentially exposed Spitak-not-treated and Gumri-not-treated groups, and the mean delta scores (change in scores) between 1½ and 25 years. At 1½ years, the mean PTSD-RI score for the Spitak-not-treated group (*M* = 53.4, s.d. = 8.4) was significantly higher than that of the Gumri-not-treated group (*M* = 43.0, s.d. = 8.9) (*t* = 6.14, df = 107, *p* < 0.001). However, at 25 years, the mean PTSD-RI scores did not differ significantly between the two groups: Spitak-not-treated group (*M* = 39.8, s.d. = 13.9) *v.* Gumri-not-treated group (*M* = 36.1, s.d. = 10.7) (*t* = 1.47, df = 107, *p* < 0.14). The mean delta PTSD-RI score for the Spitak-not-treated group (*M* = 13.7, s.d. = 15.9) was significantly greater than for the Gumri-not-treated group (*M* = 6.9, s.d. = 11.1) (*t* = 2.42, df = 107, *p* < 0.02). At 25 years, there was no significant difference between the mean DSM-5 PCL score for the Spitak-not-treated group and the Gumri-not-treated group (Table 1).

#### Depression severity at 1½ and 25 years

Table 1 shows that at 1½ years, the mean DSRS (depression) score was significantly higher for the Spitak-not-treated group (*p* < 0.001). However, at 25 years, the pattern reversed, where the mean CES-D score was less for the Spitak-not-treated group compared to the Gumi-not-treated group (*p* < 0.01).

### Gumri-not-treated and Gumri-treated groups (similarly exposed groups)

Table 2 shows the demographic characteristics, pre-earthquake and earthquake-related variables, and clinical measures for the Gumri-treated and Gumri-not-treated groups. At 1½ and 25 years there were no significant differences in the rates or mean scores for pre-earthquake, earthquake, and post-earthquake non-clinical variables between the two groups, except for mean age, with the Gumri-treated group and Gumri not-treated group (36 *v.* 38 years respectively). While age was statistically significant between the two Gumri groups, the difference was minimal, and during the initial analyses, age consistently failed to be a significant covariate. Thus, to preserve degrees of freedom, age was not included in subsequent analyses as a covariate.

#### PTSD rates at 1½ and 25 years

Table 2 also shows, between 1½ and 25 years, within-group comparisons of PTSD-RI-based PTSD rates. There was a significant decrease for both the Gumri-treated group: 33.3% to 12.1% (χ^2^ *=* 4.22, *p* < 0.04), and the Gumri-not-treated group: 35.7% to 14.3% (χ^2^ *=* 5.14, *p* < 0.02). Between-group comparison of PTSD, rates did not differ significantly at 1½ and 25-years post-earthquake.

#### PTSD severity scores (mean PTSD-RI, PCL, and delta PTSD-RI scores)

Figure 1 shows the mean PTSD-RI scores at 1½ and 25 years for the Gumri-treated group and the Gumri-not-treated group, and the delta scores between 1½ and 25 years. At 1½ years, there was no significant difference in the mean PTSD-RI scores between the Gumri-treated group (*M* = 44.0, s.d. = 9.9) and the Gumri-not-treated group (*M* = 43.0, s.d. = 8.9) (*t* = .45, df = 73, *p* = 0.66.). However, at 25 years, the mean PTSD-RI score of the Gumri-treated group (*M* = 31.1, s.d. = 10.4) was significantly lower than the Gumri-not-treated group (*M* = 36.1, s.d. = 10.6) (*t* = −2.03, df = 73, *p* < 0.05). Within-group by time (between 1½ and 25 years) comparisons of PTSD-RI scores showed a significant decrease (*p* < 0.001) for both groups. The mean delta PTSD-RI score of the Gumri-treated group (*M* = −12.9, s.d. = 11.2) was significantly greater than the mean delta score for the Gumri-not-treated group (*M* = −6.9, s.d. = 11.1) (*t* = −2.23, df = 73, *p* *<* 0.03). Also, at 25 years, mean DSM-5 PCL score was less for the Gumri-treated group compared to the Gumri-not-treated group (25.2 *v.* 33.2; *p* < 0.02) (Table 2).

#### Depression severity at 1½ and 25 years

Table 2 shows at 1½ years before treatment, that the DSRS (depression) severity scores did not differ between the Gumri-treated and -not-treated groups. At 25 years, the mean CES-D score was significantly less for the Gumri-treated group (12.6 *v.* 20.7; *p* < 0.01). At 25 years, the mean DSM-5 PCL score was lower for the Gumri-treated group compared to the Gumri-not-treated group (25.2 *v.* 33.2; *p* < 0.02).

#### Best predictors of PTSD-RI, PCL, and CES-D symptom severity scores at 25-year follow-up

Table 3 shows both the full regression model and the stepwise selected ‘best’ set of predictors contributing to the three outcome variables: (1) for PTSD-RI scores at 25 years, treatment at baseline, and higher current social support scores were predictive of lower symptom severity of PTSD-RI scores, while experiencing home destruction, higher PTSD-RI scores at 1½ years, a greater number of chronic illnesses, and worsening adversities were predictive of higher PTSD-RI scores (*R*^2^ = 36%); (2) for PCL scores at 25 years, higher social support scores and treatment were also predictive of lower PCL scores, while higher number of chronic illnesses and worsening adversities were predictive of higher PCL scores (*R*^2^ = 33%); and (3) for CES-D scores at 25 years, higher social support was predictive of lower depression, while home destruction, worsening adversities, higher number of chronic illnesses, higher SAD symptom count at 1½ years were predictive of higher CES-D score (*R*^2^ = 40%).

**Table 3.** The full model and the final stepwise model for PTSD-RI, PCL, and CES-D scores at 25-year follow-up among adults who were early-adolescents in 1988

Outcome variables and predictors	*B*	*β*	*p*	*B*	*β*	*p*
	Final model (best predictors)			Full model		
PTSD-RI score at 25 years	*F*_6, 127_ = 9.98, *p* < 0.001, *R*^2^ = 0.36			*F*_13, 121_ = 5.71, *p* < 0.001, *R*^2^ = 0.38		
1. Home destruction	−3.826	−0.146	0.055	−3.867	−0.148	0.063
2. PTSD-RI at 1½ years	0.263	0.212	0.005	0.199	0.160	0.099
3. Treatment	−4.898	−0.250	0.032	−5.175	0.174	0.027
4. Post-EQ adversities	−8.128	−0.250	0.001	−7.838	−0.241	0.001
5. Post-EQ chronic illnesses	1.877	0.224	0.003	2.229	0.266	0.001
6. Social support	−0.415	−0.265	<0.001	−0.382	−0.244	0.002
7. Sex	3.606	0.138	0.059	2.593	0.099	0.195
8. Nuclear family deaths				−1.535	−0.085	0.259
9. Severe injuries due EQ				−3.543	−0.096	0.225
10. Post-EQ trauma				0.411	0.052	0.498
11. DSRS at 1½ years				0.025	0.009	0.915
12. SAD. at 1½ years				0.611	0.111	0.172
13. Education				−1.689	−0.064	0.403
PCL score at 25 years	*F*_4, 127_ = 15.78, *p* < **0.001**, *R*^2^ = 0.33			*F*_13, 121_ = 5.56, *p* < 0.001, *R*^2^ = 0.37		
1. Home destruction				−2.245	−0.075	0.345
2. PTSD-RI at 1½ years	−6.542	−0.192	0.009	0.096	0.068	0.486
3. Treatment	−5.186	−0.140	0.056	−5.194	−0.153	0.053
4. Post-EQ adversities	3.396	0.354	<0.001	−5.120	−0.138	0.064
5. Post-EQ chronic illnesses	−0.526	−0.294	<0.001	3.482	0.364	<0.001
6. Social support				−0.501	−0.280	<0.001
7. Sex				1.294	0.043	0.572
8. Nuclear family deaths				−1.230	−0.059	0.430
9. Severe injuries due EQ				−4.119	−0.098	0.220
10. Post-EQ trauma				1.004	0.111	0.150
11. DSRS at 1½ years				−0.115	−0.039	0.664
12. SAD at 1½ years				0.652	0.104	0.205
13. Education				−1.047	−0.035	0.651
CES-D score at 25 years	*F*_6, 128_ = 14.08, *p* < **0.001**, *R*^2^ = 0.40			*F*_13, 121_ = 6.94, *p* < 0.001, *R*^2^ = 0.43		
1. Home destruction	−3.160	−0.131	0.065	−2.537	−0.105	0.169
2. PTSD-RI at baseline	−6.785	−0.226	0.002	−0.131	−0.114	0.222
3. Treatment	2.798	0.362	<0.001	−3.272	−0.119	0.114
4. Post-EQ adversities	−0.420	−0.290	<0.001	−7.068	−0.235	0.001
5. Post-EQ chronic illnesses	0.880	0.120	0.093	2.798	0.362	<0.001
6. Social support	0.791	0.155	0.026	−0.428	−0.296	<0.001
7. Sex				0.467	0.019	0.792
8. Nuclear family deaths				1.316	0.079	0.276
9. Severe injuries due EQ				−0.808	−0.024	0.755
10. Post-EQ trauma				0.754	0.103	0.162
11. DSRS at 1½ years				0.075	0.031	0.715
12. SAD at 1½ years	0.791	0.155	0.26	0.931	0.183	**0.020**
13. Education				−1.85	−0.076	0.302

PTSD-RI, Posttraumatic Stress Disorder-Reaction Index; PCL, PTSD Checklist; CES-D, Center for Epidemiologic Studies-Depression; EQ, earthquake; SAD, separation anxiety disorder.

## Discussion

To our knowledge, this is the first long-term (past 5 years) prospective study examining the 25-year status of PTSD and depressive symptoms, risk and protective factors, and treatment outcome among adults who experienced a catastrophic earthquake as early adolescents. This study culminates a 30-year series of initial and follow-up studies among children, adolescents, adults, and older adults emanating from a major post-disaster mental health outreach, recovery, and research program implemented in the aftermath of the catastrophic 1988 Spitak earthquake in Armenia. The clinical findings of importance include the chronicity of PTSD and depressive symptoms 25 years after the disaster among a substantial portion of the exposed population, and the suggested long-term benefits of trauma-focused therapy for early adolescents. Of note, the school-based intervention used at 1½ years post-earthquake was a precursor of the UCLA Trauma Psychiatry Program trauma/grief intervention that has evolved into Trauma and Grief Component Therapy for Adolescents (Saltzman et al., 2017).

### Exposure effect: comparison of not-treated groups of Gumri (moderate–severe exposure) and Spitak (very severe exposure)

At 1½ years, PTSD rates and mean severity scores based on PTSD-RI were higher in the Spitak-not-treated group compared to the Gumri-not-treated group, commensurate with the rates of mortality (Spitak = 37%; Gumri = 28%), morbidity (Spitak = 23.1%; Gumri = 2%), and home destruction (Spitak = 83%; Gumri = 50%). At 25-year follow-up, rates of PTSD and mean severity scores subsided significantly in both groups, with the Spitak group manifesting a greater decrease in PTSD-RI scores (*p* < 0.02). The regression analysis indicated that adversities were risk factors for both the PCL and PTSD-RI scores. The post-earthquake adversities were significantly less for the Spitak group (*p* < 0.03). This was consistent with citywide faster recovery in Spitak (e.g. more reconstruction), with lower rates of poverty (36% Spitak *v.* 44% Gumri), and lower unemployment (37% Spitak *v.* 47% Gumri) (Goenjian, Khachadourian, Armenian, Demirchyan, & Steinberg, 2018; Statistical Committee of the Republic of Armenia, 2015). The greater decline of PTSD severity scores among the Spitak participants may be attributable in part to the fewer post-earthquake adversities experienced by the Spitak group.

The differential effect of trauma on adults *v.* children and adolescents is an important topic that has not been investigated adequately in the literature. Comparison of the mean PCL scores, from a prior 23-year follow-up study of survivors from Gumri and Spitak who were adults at the time of the earthquake (Goenjian et al., 2018), with the PCL scores of the not-treated subjects from Spitak and Gumri of the current study at 25-years, indicates a differential effect of age at time of exposure. Those who were early-adolescents at time of exposure showed significantly higher mean PCL scores at follow-up (Spitak *M* = 31.5; Gumri *M* = 33.2) compared to those who were adults at the time of exposure (Spitak *M* = 25.3; Gumri *M* = 19.7) (*p* < 0.01 for both cites) suggesting that adolescents may be more vulnerable than adults to developing chronic PTSD symptoms and should be prioritized for early risk screening and treatment. The age effect of trauma on long-term sequelae needs further investigation.

Regarding depression, at 1½-years, the DSRS score for the Spitak-not-treated group was significantly higher compared to the Gumri-not-treated group. However, at 25-years these scores were reversed. For the Spitak-not-treated group, with fewer post-earthquake adversities (*p* < 0.03), the mean CES-D score was lower than that of Gumri-not-treated group. Similar to PTSD, fewer adversities experienced and the more favorable recovery environment, including more rebuilding, employment, and less poverty in Spitak may have contributed to the faster recovery of depression symptoms.

### Treatment effect: comparison of Gumri-treated *v.* Gumri-not-treated groups

At 1½ years, there were no significant differences in pre-, peri-, and post-earthquake variables, including PTSD-RI severity scores and PTSD rates between the Gumri-treated and Gumri-not-treated groups. A treatment effect was evident at 25-years post-earthquake, with the mean PTSD-RI and PCL scores for the Gumri-treated group being significantly lower than the Gumri-not-treated group. PTSD-RI scores had decreased in both groups at follow-up; however, the decrease was significantly greater for the Gumri-treated group (30% *v*. 16%). The treatment effect was consistent with the regression analyses showing that treatment at baseline was predictive of lower PTSD symptom severity at follow-up.

Several aspects of the intervention lend themselves as possible candidates for the long-term benefits of treatment. First, the focus in treatment on identifying and managing trauma reminders may have led to the reduction of reminder-related reactivity. Second, treatment promoted strategies for coping with hyperarousal and avoidant behavior. Finally, the promotion of skills for managing emotions and recruiting social support may have been associated with less risk-taking behavior, and anger outbursts.

CES-D scores at 25-years also showed a treatment effect, with significantly lower CES-D scores for the Gumri-treated group. Treatment included dealing with losses, enhancing problem-solving of adversities, reducing interpersonal conflicts, and promoting adaptive rather than maladaptive grief reactions. These components may have contributed to the reduction in depressive symptoms.

### Risk and protective factors for PTSD and depression

There were two risk factors (post-earthquake adversities and chronic illnesses), and two protective factors (treatment and current social support) contributing to the variance in PTSD-RI, PCL, and CES-D scores at 25 years. The present findings extend shorter-term findings after disasters, indicating that adversities constitute a risk factor in adults for PTSD (Brewin, Andrews, & Valentine, 2000; Galea, Nandi, & Vlahov, 2005). Adverse conditions in the earthquake zone included lack of heat, electricity, housing, transportation, and destruction of physical and community infrastructure. Even though the Gumri-not-treated group had experienced less destruction and lower PTSD-RI scores at 1½ years compared to Spitak, post-earthquake adversities experienced were reported to be greater by the Gumri subjects, and the decrease in PTSD-RI scores among the Gumri-not-treated subjects was significantly less compared to the Spitak-not-treated group. The fewer adversities reported by the Spitak subjects was congruous with the city-wide faster rebuilding, lower unemployment, and poverty. This finding suggests that recovery programs include attention to alleviation of post-disaster adversities. In so doing, governmental and non-governmental agencies may play an important role in the mental health recovery of survivors. Improvements in these areas can foster social interaction (e.g. visits to relatives, participation in community activities), and enhance the social ecology.

The number of post-earthquake chronic illnesses was predictive of PTSD and depressive scores at 25 years. This is the first study documenting the association of chronic illness with PTSD and depression among survivors over a decade after a disaster. These results extend the findings of a 4-year follow-up study among adult survivors of the Spitak earthquake that found an increase in heart disease, newly reported hypertension, diabetes, and arthritis (Armenian, Melkonian, & Hovanesian, 1998). The finding of long-term health conditions among adolescents exposed to a disaster expands current discussions of the relationship between adverse childhood experiences and health outcomes (Felitti & Anda, 2010).

Higher levels of current social support were inversely related to PTSD-RI and CES-D scores at 25 years. The association of PTSD symptoms and social support extends prior findings among adults in this population (Goenjian et al., 2018) and children after other disasters (McDermott, Berry, & Cobham, 2012; Thordardottir et al., 2016; Udwin et al., 2000). Given that current levels of social support may have been indicative of earlier levels of social support over the post-earthquake interval, we would suggest, as has been previously reported (Kaniasty & Norris, 2008; Platt, Lowe, Galea, Norris, & Koenen, 2016), that there may be a bi-directional relation, in that ongoing social support may have contributed to the resolution of PTSD symptoms, while ongoing severity of PTSD symptoms may have negatively affected the acquisition and maintenance of social support. The bi-directionality may also apply to the association of social support and depression.

SAD symptoms at baseline also predicted depression at 25 years. Many of the early adolescents in the aftermath of the earthquake were fearful of something bad happening again or to their caretakers and avoided separation from them by refusing to go to school (Goenjian et al., 1995). Genetic vulnerabilities may also play a partial explanatory role. A multigenerational family study from Gumri after the earthquake found a significant genetic correlation between anxiety symptoms and depression (*r*_g_ = 0.54) indicating pleiotropy (shared genetic vulnerability) (Goenjian et al., 2008). This shared genetic vulnerability may have contributed to the predictive value of SAD in predicting depression.

### Strengths

Strengths of the study included the extensive long-term follow-up of exposed preadolescents with 87% of the original cohort retrieved at follow-up, exposure of all of the subjects to the trauma contemporaneously, and the inclusion of treated and not-treated subjects. Additionally, we used the same instrument, the DSM-III-R based PTSD-RI scale, to measure symptom severity at 1½ and 25-year follow-up to reduce discrepancies introduced by using different measures at different time points. Finally, we used the PCL to estimate current rates of PTSD based on DSM-5 criteria.

### Limitations

The response to some of the questions dating back 25 years, such as the distress experienced during the earthquake and post-earthquake adversities may have been subject to recall bias. Given that children were not randomly assigned to treatment, confidence in the outcome findings must be qualified in that there may have been unknown differences in the comparison groups.

### Implications for post-disaster public mental health recovery programs

Despite the decline, a sub-group of the population in both cities continued to experience substantial levels of PTSD and depressive symptoms. This represents a public mental health problem for the region. In addressing chronic cases, mental health programs should include ongoing surveillance for PTSD and depression, as well as the post-earthquake onset of traumatic experiences. Public health programs should also strive to mitigate ongoing post-disaster adversities.

The association of persistent PTSD and depression with chronic medical illnesses point to the need for targeted outreach services across physical and behavioral health systems. Finally, the enduring impact of treatment provided at 1½ years post-earthquake reinforces the benefit of implementing school-based trauma-grief focused interventions that may benefit a generation of adolescents as they transition to adulthood.
